# APTES-Modified Nanocellulose as the Formaldehyde Scavenger for UF Adhesive-Bonded Particleboard and Strawboard

**DOI:** 10.3390/polym14225037

**Published:** 2022-11-21

**Authors:** Jakub Kawalerczyk, Joanna Walkiewicz, Dorota Dziurka, Radosław Mirski, Jakub Brózdowski

**Affiliations:** 1 Department of Mechanical Wood Technology, Faculty of Forestry and Wood Technology, Poznan University of Life Sciences, 60-627 Poznan, Poland; 2 Department of Chemical Wood Technology, Faculty of Forestry and Wood Technology, Poznan University of Life Sciences, 60-627 Poznan, Poland

**Keywords:** nanocellulose, silanization, urea-formaldehyde adhesive, strawboard, particleboard

## Abstract

This work examines the possibility of applying non-modified nanocellulose and nanocellulose functionalized with 3-aminopropyltriethoxysilane (APTES) as a formaldehyde scavenger for commonly used urea-formaldehyde (UF) adhesive. The effect of silanization was determined with the use of Fourier transform infrared spectroscopy (FTIR), flame atomic absorption spectrometry (FAAS), and elemental analysis. Moreover, the ability of cellulosic nanoparticles to absorb the formaldehyde from an aqueous solution was investigated. After homogenization, cured UF adhesives were examined with the use of FTIR, energy-dispersive spectroscopy (SEM-EDS), and the perforator method to determine the content of formaldehyde. Manufactured boards made of rape straw particles and wood particles were tested in terms of their physico-mechanical properties and formaldehyde emission. Studies have shown that the applied method of silanization was effective. Furthermore, in the case of non-modified nanocellulose, no sign of formaldehyde scavenging ability was found. However, the functionalization of cellulosic nanoparticles with APTES containing an amino group led to the significant reduction of formaldehyde content in both the aqueous solution and the UF adhesive. The mechanical properties of both strawboards and particleboards were improved due to the nanocellulose reinforcement; however, no effect of silanization was found. Nevertheless, functionalization with APTES contributed to a decrease in formaldehyde emission from boards, which was not found in the case of the introduction of non-modified cellulosic nanoparticles.

## 1. Introduction

Formaldehyde-containing adhesives account for approximately 95% of all wood adhesives used in the production of wood-containing composites and 85% of them are UF resins [1]. They owe their popularity to a number of advantageous properties, such as high reactivity, lack of color, competitive price, and good performance in indoor environments. However, one of the greatest disadvantages of UF resins is the hazardous emission of formaldehyde. Their application may cause indoor air pollution and potential health issues because of the free formaldehyde emitted from the UF-bonded wood-based panels [2,3]. Substances such as formaldehyde, acetone, acetaldehyde, and ethyl-1-hexanol were found to be the main pollutants detected in indoor residential environments. These substances were also closely related to the occurrence of mucosal symptoms among the examined occupants [4,5]. Many studies, performed both in vitro and in vivo, confirmed the toxic effects on various human or animal cells and organs such as the lung, upper respiratory tract, brain, and bone marrow in cases of formaldehyde occupational exposure [6,7]. Taking into account the emerging reports on health hazards, the International Agency for Research on Cancer (IARC) re-classified formaldehyde from a “probable human carcinogen” to a “known human carcinogen” in 2004 [8,9]. Its emission from UF resins is mostly sourced from the unreacted formaldehyde, hydrolytic degradation of bond lines under moisture conditions, and the reversibility of aminomethylene link in the cured adhesive network [10,11,12]. One of the methods contributing to the reduction of formaldehyde emission from wood-based products is the introduction of proper additives, so-called formaldehyde scavengers, to the resins. These can be various types of substances such as chemicals, mineral particles, particles rich in phenolic substances, particles with high protein content, nanoparticles, etc.

Formaldehyde scavengers in general can be defined as substances characterized by the ability to bond free formaldehyde, which can be used in both post-treatment and as a modifier added to the resin during homogenization [8,13]. In the case of UF resins, efforts are still being made to find such additives that reduce harmful emissions and, at the same time, do not deteriorate the mechanical and physical properties of the resultant wood-based boards. An interesting example of an additive applied for UF resins to improve their strength properties is cellulose derivatives. Among them, microcellulose (MC) and nanocellulose (NC) are the most commonly studied as reinforcing agents for amino resins (Table 1). The reasons for such scientific attention focused on NC in recent years are its unique properties such as low density, high-energy conversion capacity, exceptional mechanical strength, tremendous surface area, and chemical compatibility [14,15,16].

In general, it is difficult to analyze and compare the results of formaldehyde emission from wood-based panels because it depends strongly on the pressing parameters, the type of adhesive applied, and the test method [26]. Therefore, based on the previously conducted studies, it cannot be determined whether the reduction of formaldehyde emission resulted from the addition of nanocellulose itself due to its reactive nature or, for example, increased strength of glue joints or improved morphology of the glue line. Usually, the available literature sources do not focus on the reason behind the decreased formaldehyde emission, which is especially interesting since different studies show different conclusions. However, studies have indicated that the introduction of silane-modified cellulose derivatives can improve their effectiveness in lowering formaldehyde emissions when compared with non-modified ones. Park and Causin [27] stated that the incorporation of amine derivatives is one of the most effective methods for reducing formaldehyde emissions from UF adhesives. Moreover, according to Neves et al. [28], silanization is the most frequently applied treatment of cellulose in studies on polymer reinforcement. It improves the dispersion and interfacial adhesion between the polymer and the filler [29].

Table 1 also shows that no research has been carried out so far on the application of a nanocellulose-modified UF adhesive in the production of strawboards. Due to the continuous shortages of wood, increasing prices, and growing competition for raw materials between industries, there is a demand for alternative lignocellulosic materials for board manufacturing [30]. According to Dziurka and Mirski [31], the most promising concept is to use straws because of their availability. In Poland, the annual production of straws is estimated at 25–28 million tons [32]. Moreover, rape straw was already found to be a valuable raw material for the production of various types of panels such as structural boards [33], lightweight boards for furniture making [34], insulation boards [35], boards for use in outdoor conditions [36], etc. Furthermore, it is expected that, due to many factors, research focused on the use of alternative lignocellulosic particles for the production of boards will continue to expand intensively in the future [37].

Therefore, considering that emerging scientific reports on the influence of nanocellulose on formaldehyde emission are still inconclusive and there is a lack of knowledge on using nanocellulose-reinforced adhesives in strawboard production, the present study aimed to investigate the effect of both non-modified and APTES-modified nanocellulose introduction to the UF resin on the properties of boards made of rape straw particles and wood particles. Additionally, based on the presented research, it will be possible to determine whether the use of UF resin reinforced with a small amount of nanocellulose will allow for the production of rape strawboards with equally good properties as commonly used particleboards.

## 2. Materials and Methods

### 2.1. Materials

Commercially available UF adhesive was purchased from the market with the following characteristics: Viscosity of 470 mPa × s, solid content of 58%, gel time of 88 s at 100 °C, and pH of 8.11. The nanocellulose with the trade name NG01NC0101-1000 was supplied by Nanografi Laboratory (Ankara, Turkey). Nanoparticles were obtained by sulfuric acid hydrolysis. They were characterized by an average particle width of 10-20 nm and length of 300–900 nm as confirmed by the producer based on transmission electron microscopy (TEM) images. The 3-aminopropyltriethoxisilane (99% pure) was supplied by Merck (Poznan, Poland). The ammonium nitrate introduced as a hardener (20%wt) was purchased from Chempur (Piekary Slaskie, Poland). Pine (*Pinus sylvestris* L.) wood particles intended for the production of the middle layer of particleboard in industrial conditions were supplied by the manufacturer of wood-based panels. Rape straw (*Brassica napus* L. var. napus) was ground in a disc chopper to obtain particles with a similar length to wood particles.

### 2.2. Nanocellulose Silanization

**Figure 1:**
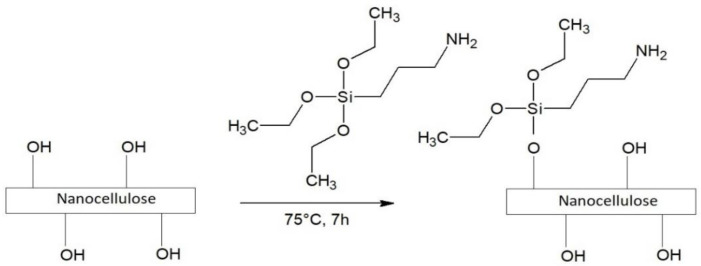
Schematic diagram of APTES-modified nanocellulose preparation.

The process of silanization with APTES (Figure 1) was carried out using the reaction conditions proposed by Wang et al. [38]. In order to obtain the mixture of ethanol and water, 64 mL of ethanol was dissolved in 16 mL of deionized water. Thereafter, 16 ± 0.01 g of APTES and 4 ± 0.01 g of nanocellulose were added to a 200 mL flask. In order to increase the effectiveness of silanization, the glacial acetic acid was slowly added to the solution as a catalyst until the pH reached 4 as recommended by Neves et al. [39]. Then, the mixture was stirred vigorously with the magnetic stirrer at 75 °C for 7 h. The APTES-modified powder was filtered and washed with distilled water. Following the observations of Siuda et al. [40], drying the obtained samples in a laboratory oven at a high temperature was abandoned. Instead, APTES-modified nanocellulose was dried in the desiccator above the silica gel for 72 h at room temperature to increase the effectiveness of silanization.

### 2.3. Characterization of Modified Nanocellulose

Samples of non-modified and APTES-modified NC were marked as NC-N and NC-S, respectively. In order to evaluate the effect of silanization, FTIR (Fourier transform infrared spectroscopy) analysis was used. The NC powder was mixed with KBr (1/200 mg ratio), and the spectra were registered with an Alpha FTIR spectrometer (Bruker Optics GmbH, Ettlingen, Germany) with the Fourier transform range of 400–4000 cm^−1^, registering 16 scans at a resolution of 4 cm^−1^. Moreover, the total crystallinity index (TCI, A1370/A2900) and the lateral order index (LOI, A1430/A898) were determined according to Nelson and O’Connor [41].

The concentration of silicon in samples was investigated with the use of flame atomic absorption spectrometry (FAAS). First, 0.5 g of samples were mineralized using nitric acid (8 mL) in a semi-closed microwave mineralization MARSX pressing system (CEM Corporation, Germany). After mineralization was completed, the samples were then filtered and diluted with deionized water to 50 mL. The analysis of Si content in samples was performed with the use of the Spectra 280 AA spectrometer (Agilent Technologies, Santa Clara, CA, USA). The calibration curve was obtained from the serial dilutions of the Si standard solution. The average values of three simultaneous measurements were presented as a final result.

The concentration of nitrogen in samples was analyzed with a Flash 2000 elemental analyzer (Thermo Fisher Scientific, Waltham, MA, USA). The equipment was calibrated with 2,5-bis-(tert-butyl-benzoxazole-2-yl)thiophene (Thermo Fisher Scientific, Waltham, MA, USA) and certificated materials such as birch leaf and Alfalfa (Elemental Microanalysis Ltd., Okehampton, UK). The six-point calibration curve was plotted with the use of the K factor. The results were expressed as the average values of measurements performed three times.

An aqueous solution of formaldehyde (0.1 M) was prepared to investigate the ability of APTES-modified and non-modified nanocellulose powders to absorb the formaldehyde. To prepare 0.1 M formaldehyde solution, 5 drops of sulphuric acid were mixed with 100 mL of 15% formaldehyde solution and then refluxed to depolymerize the paraformaldehyde for 15 min. After cooling was completed, the solution was neutralized to pH 7 with NaOH and diluted in distilled water. Then, the assumed amount of sample was introduced to the solution. The prepared formaldehyde and water solutions containing NC were kept in the laboratory oven at 65 °C for 3 h. The solution was then filtered, and the content of formaldehyde was determined with the use of sodium sulfite titration. Both 25 mL of 0.1 M Na_2_SO_3_ and 2 mL of 0.1 M NaOH were added to a 10 mL aliquot of the filtrate containing formaldehyde. The titration was performed with the 0.05 M HCl solution and phenolphthalein as an indicator. A reference analysis was carried out with the same procedure excluding the formaldehyde. Furthermore, the analysis of the formaldehyde solution incubated for 3 h at 65 °C without any additives was also conducted. Three repetitions were performed for each solution. This method was previously used to analyze the ability to reduce the formaldehyde content in an aqueous solution via the addition of lignocellulosic sludge [42], bark powder [43], and soy flour [44].

### 2.4. Adhesive Mixture Preparation

There is a necessity to process cellulosic nanoparticles in a wet state to achieve proper dispersion, therefore, a 10% aqueous NC suspension was mixed with the magnetic stirrer at 700 rpm for 10 min. The amount of the introduced suspension was adjusted so that the adhesives contained 1% or 2% of nanocellulose. It was adjusted in order to develop variants suitable for the production of wood-based boards. According to Veigel et al. [45], the amount of introduced NC is crucial to achieving the optimum reinforcement effect in the case of a UF adhesive. The authors concluded that taking into account the highly hydrophilic nature of cellulose itself, the amount of nanoparticle introduced should be in the range of 1–3%. The viscosity of the mixtures must be low enough to ensure the proper application of the adhesive on the wood surface. Moreover, nanoparticles in general have a tendency to assemble around each other and form agglomerates. Thus, too much nanocellulose introduced to the adhesive can cause deterioration of the bonding strength due to the concentration of stress at certain points (agglomerates) in the glue line [46]. The applied compositions of both modified and reference adhesive mixtures are shown in Table 2.

After the addition of components, the mixtures were subjected to the mixing process at 1000 rpm for 2 min using the CAT-500 homogenizer (Ingenieurburo CAT, M.Zipper GmH, Ballrechten-Dottingen, Germany) to attain the proper level of homogenization.

### 2.5. Investigations of Cured Adhesive Properties

After the adhesives were cured in the laboratory oven at 130 °C and ground using a laboratory mill, the obtained powder characterized by a dimensional fraction of 0.125 × 0.125 mm^2^ was used to perform the analysis. The cured powder was mixed with KBr at a 1/200 mg ratio. Spectra were registered with the Alpha FTIR spectrometer (Bruker Optics GmbH, Ettlingen, Germany) with the Fourier transform range of 400–4000 cm^−1^, registering 16 scans at the resolution of 4 cm^−1^.

**Figure 2:**
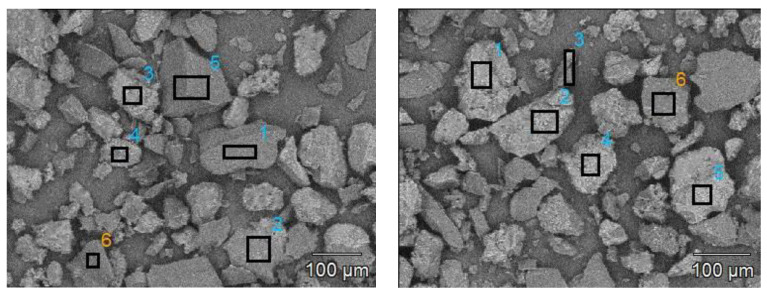
The selection of areas for silicon content measurements (numbers mark the areas selected for analysis).

The SEM-EDS method was used to determine Si content in the cured UF mixtures. The representative amount (10 ± 0.1 mg) of adhesive powder was sprinkled onto the double-sided carbon tape (5 × 20 mm^2^) mounted on the SEM stub. Each sample was analyzed by randomly selecting 6 fields on individual resin particles (Figure 2). The Si content was determined within those areas using the scanning electron microscope Hitachi SU3500 working at the accelerating voltage of 15 kV.

The formaldehyde content in cured adhesives was determined in accordance with the assumptions of the perforator test EN 120 [47]. It is a well-established method, commonly used to determine the formaldehyde content from wood-based boards. However, in order to investigate the formaldehyde content in the adhesive powder, the method was adjusted and modified following the guidelines proposed by Dziurka and Mirski [48]. First, 5 ± 0.01 g of ground adhesive powder was subjected to toluene extraction in the perforator apparatus. Formaldehyde content in the obtained aqueous solution was investigated by spectrophotometry using the commonly applied ammonium acetate and acetylacetone method. The absorbance of the samples was determined on a Biosens UV-5600 spectrophotometer (Biosens, Warsaw, Poland) at 412 nm. The analysis of each adhesive variant was performed in five replicates.

### 2.6. Boards Manufacturing and Testing

**Figure 3:**
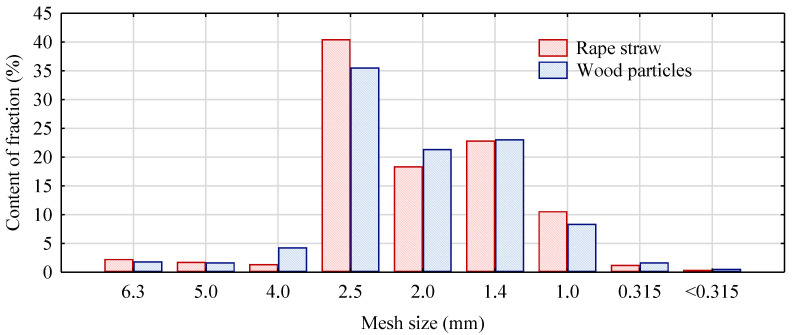
Fractional composition of rape straw particles and wood particles.

To characterize the dimensions of wood and rape straw particles, their fractional composition was determined using sieve analysis. It was conducted with the use of flat sieves possessing mesh with square perforations of 6.3, 5.0, 4.0, 2.5, 2.0, 1.4, 1.0, and 0.315 mm. The results are presented in Figure 3. Based on the obtained outcomes, it was found that both wood and straws were characterized by a similar fractional composition. The dimensions of the majority of particles ranged between 1.4 and 2.5 mm.

Materials were dried at 110 °C to reach the moisture content of 2 ± 2%. The gluing degree (ratio of the dry mass of the adhesive to the dry mass of wood or straw) was 8%. For each variant of adhesive presented in Table 2, two single-layer strawboards and two single-layer particleboards with an assumed thickness of 15 mm and a density of 600 kg/m^3^ were prepared. The pressing process was conducted at 180 °C with a unit pressure of 2.5 N/mm^2^ for 20 s/mm of the final board thickness.

After conditioning for 7 days at the relative humidity of 65 ± 5%, 20 ± 2 °C, the mechanical properties of manufactured panels such as their bending strength, modulus of elasticity, and internal bond were investigated according to the relevant standards, namely, EN 310 [49] and EN 319 [50], respectively. Moreover, the density was evaluated according to EN 323 [51]. In order to determine the water resistance of the boards, thickness swelling was investigated after 2 and 24 h according to EN 317 [52]. The determinations of mechanical and physical properties were performed using 12 samples from each variant of boards. The formaldehyde emission was analyzed three times for each variant with the use of gas chamber analysis according to EN ISO 12460-3 [53] using a GreCon GA 6000 analyzer (Fagus-GreCon Greten GmbH & Co. KG., Alfeld, Germany). Formaldehyde content in an aqueous solution was determined spectrophotometrically with the use of the ammonium acetate and acetylacetone method. Absorbance was measured on a Biosens UV-5600 spectrophotometer at 412 nm (Biosens, Warsaw, Poland).

### 2.7. Statistical Analysis

The obtained results were subjected to multivariate statistical analysis ANOVA. Moreover, in order to distinguish homogeneous groups and evaluate the significance of observed changes, the Tukey test on the significance level of α = 0.05 was performed using a Statistica 13.0 software (StatSoft Inc., Tulsa, OK, USA).

## 3. Results and Discussion

**Figure 4:**
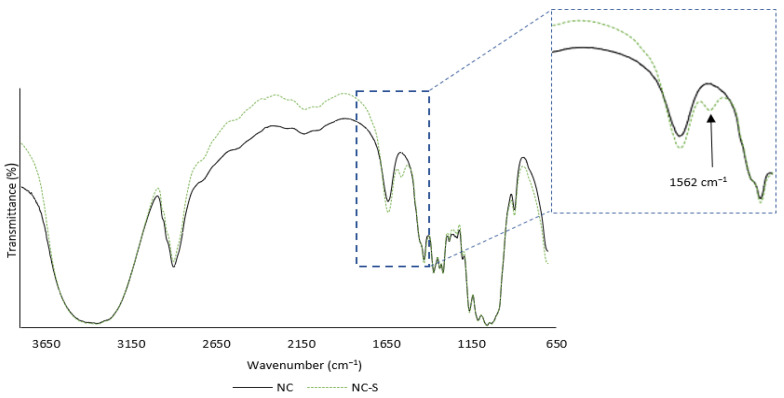
The FTIR spectra of non-modified and APTES-modified nanocellulose.

FTIR spectra of non-modified NC and salinized NC are presented in Figure 4. The broad peak at 3000–3550 cm^−1^, which can be assigned to O–H groups and the intracellular hydrogen bonding of cellulose, was observed [54]. C-H stretching vibrations were noted at 2830–2975 cm^−1^ [55]. The peak absorption observed at 1638 cm^−1^ was assigned to the O-H vibration of water in the structure of the NC [56,57] and the peaks, which can be noted from 1315 to 1373 cm^−1^, are shown because of the bending vibration of the C–H and C–O groups in the polysaccharide rings of cellulose [57,58]. The peak observed at 1053 cm^−1^ was associated with the bending vibration of C–O–C and the cellulose chains are observed around 897 cm^−1^ [59]. The peaks listed above appear for nanocellulose and silanized nanocellulose samples. However, in the case of samples modified with APTES, a peak at 1562 cm^−1^ can be noted. This peak corresponds to bending vibrations of -NH_2_ groups present in the APTES structure [60]. Moreover, C–O bending modes contributed to the overlapping of peaks derived from Si-O-Si bonds of the silicon compounds [60]. The indication of the presence of amino groups is particularly beneficial taking into account the potential application of NC as a formaldehyde scavenger because the -NH_2_ group located on top of the APTES molecule may potentially react with the formaldehyde in UF resin [25].

According to Tang et al. [61], the crystallinity of nanocellulose is one of the crucial factors affecting its mechanical and thermal properties. The results of the calculated indexes are presented in Table 3. The lateral order index (LOI) was similar regardless of the variant of nanocellulose. However, in the case of the total crystallinity index (TCI) of NC-S, a slightly lower value was found. The same effect of decreased crystallinity was observed in previously conducted studies regarding polymer reinforcement with silane-modified NC [62,63]. The reason could be the fact that the surface of nanocellulose was grafted with the amorphous silane side chains, which consequently could lead to the reduction in TCI [63].

The analysis of silicon and nitrogen concentrations in cellulose is a commonly applied method allowing the determination of silanization effectiveness [64]. The concentrations of both N and Si are presented in Table 4. Based on the outcomes, it was found that the APTES-modified NC was characterized by a high concentration of nitrogen and silicon. On the other hand, the presence of these elements was not observed in the non-modified variant. The increased content of silicon from the -Si-(O-CH_2_-CH_3_)_3_ groups and nitrogen from -NH_2_ groups may indicate that the applied method of treatment with APTES was effective. Moreover, the obtained values were higher than those presented by Neves et al. [65] for APTES-functionalized microcellulose. The reason could be the increased chemical reactivity of nano-sized particles compared with the cellulosic derivatives characterized by the larger particle sizes [66]. Based on FTIR spectra and elemental analysis, it can be assumed that APTES was bonded to cellulosic nanoparticles. However, due to the chemical structure, not all hydroxyl groups of nanocellulose are highly reactive. According to observations presented in the literature, the attachment of modifying molecules takes place mainly at C6 and C2 and results from regioselectivity [67,68]. Moreover, Beaumont et al. [69] reported that organosilicon groups such as mercapto-, vinyl-, and azido- functionalities were attached to the cellulose at the C6 position. Therefore, it can be assumed that a similar mechanism occurred during the present functionalization

**Figure 5:**
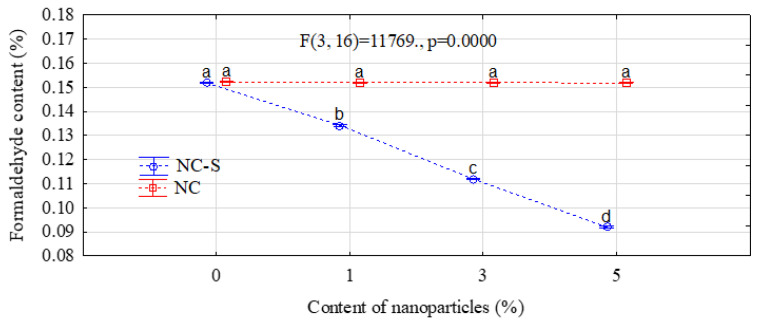
The ability of NC and NC-S to absorb the formaldehyde (letters a, b, c and d mark homogeneous groups in HSD Tukey test).

The results of the formaldehyde-scavenging ability of NC and NC-S are presented in Figure 5. Based on the investigations, it was found that non-modified NC did not show the ability to absorb formaldehyde. Regardless of the amount of nanoparticle introduced, the changes were not statistically significant. There are many reports in the literature about the positive effect of nanoparticles on the emission of formaldehyde even at a very low loading level. It is usually attributed to their absorbability, shielding effect, high reactivity, and barrier properties [70,71,72]. However, none of these effects have been observed in this study for non-modified NC. On the other hand, the modification of cellulosic nanoparticles with the use of silane resulted in a significant improvement in the ability to absorb formaldehyde. As the amount of NC-S increased, the formaldehyde content in the aqueous solution noticeably decreased by up to 39% in the case of the highest load of nanoparticles. This effect was most likely caused by the introduction of amino groups contained in the APTES molecule. According to Nomura and Jones [73], the occurrence of amine groups can contribute to the decrease in formaldehyde content due to bonding with formaldehyde.

**Figure 6:**
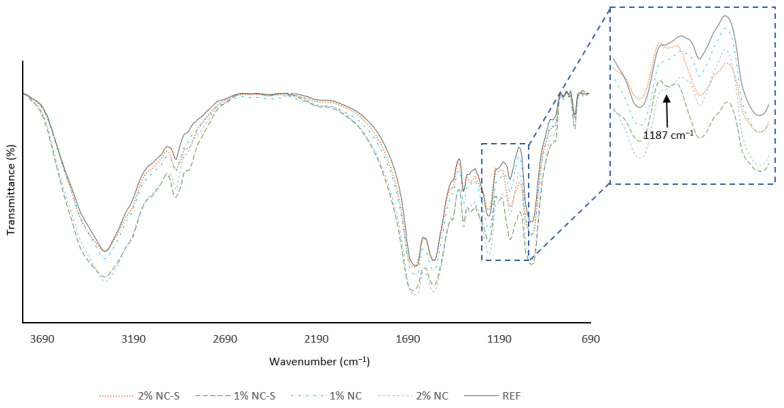
FTIR spectra of adhesive powders.

FTIR spectra of UF resin with the addition of NC and NC-S are presented in Figure 6. The spectra mostly revealed peaks characteristic of UF resin. Peaks corresponding to O–H groups (3356 cm^−1^) and the stretching vibrations of C–H (2956 cm^−1^) were observed. Moreover, the peaks at 1646 cm^−1^ and 1540 cm^−1^ were assigned to C=O stretching and N–H bending bands in amides I and II [74,75]. The C–N stretching and N–H bending vibrations of amide III were observed at 1246 cm ^−1^ [76]. Moreover, in the case of silane-modified samples, a peak at 1187 cm^−1^ occurred and was attributed to the presence of siloxane groups (Si-O-Si) [77,78].

The results of silicon content measurements in the cured adhesive powder are summarized in Table 5. The results have shown that the Si content increased with the increasing addition of modified nanocellulose. The small level of standard deviations is also a valuable observation because it may indicate good homogenization of the resin mixture and good dispersion of nanoparticles within the adhesive matrix. Usually, a large number of hydroxyl groups on the surface of nanocellulose results in poor dispersion, which can negatively affect the result [79]. Therefore, silanization could contribute to the improvement in particle dispersion by making the NC molecules more hydrophobic [80]. This is especially important because the level of NC dispersion in the polymer has a significant effect, for example, on its strength properties [63]. The outcomes of many previously conducted studies focused on the reinforcement of polymer composites confirmed that decreasing the hydrophilicity of NC prior to blending with polymers resulted in the enhancement of their mechanical properties [81,82,83,84].

**Figure 7:**
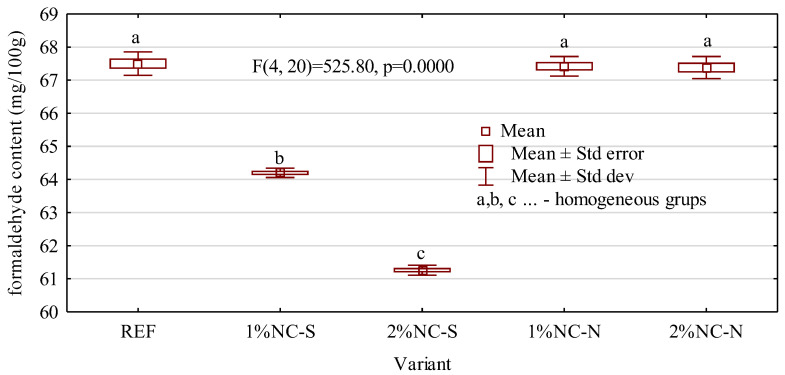
Formaldehyde content in cured adhesive.

Based on the conducted analysis, it was found that the addition of non-modified nanocellulose did not reduce the amount of formaldehyde contained in the adhesive (Figure 7). There were no statistically significant changes when compared to the reference variant. However, the introduction of APTES-modified cellulosic nanoparticles led to a significant decrease in formaldehyde content. It was reduced by 5% and 10% due to the reinforcement with 1% and 2% silanized nanocellulose, respectively. Therefore, the observed effect showed the same tendency as in the case of the ability to absorb the formaldehyde from an aqueous solution. The reason for the reduction is likely the fact that amines can undergo a reaction with formaldehyde and form methylol groups, which in turn can form methylene bridges due to further reactions [25,85,86]. This effect is particularly beneficial since it can indicate that nanocellulose modified with APTES can contribute to the decrease in the amount of hazardous emission from the UF adhesive-bonded materials.

The effect of UF resin modification on the properties of manufactured rape strawboards and particleboards is shown in Table 6. It was found that regardless of the variant, the modification had no effect on the final density of the boards. The comparison of the reference variants showed that the boards made of rape straw were characterized by lower strength than the particleboards. This could have been caused by differences in the chemical composition of raw material since rape straw contains less cellulose and lignin than pine wood, and there are indications in the literature that this can potentially influence the mechanical properties of the boards [31,87,88]. Moreover, straws in general are characterized by the presence of waxes on their surface, which could adversely affect gluing and adhesion [89]. The UF resin modification with nanocellulose had a significant effect on the strength properties of manufactured boards. It caused an improvement in the bending strength, modulus of elasticity, and internal bond of panels made of both wood particles and rape straw. However, no statistically significant influence of the amount of added modifier and functionalization with APTES was noted. In the case of particleboards, the improvement was significant enough to change the classification of the board from P1 (general-purpose boards for use in dry conditions) to P2 (boards intended for interior application for use in dry conditions) according to EN 312 [90]. Furthermore, the outcomes of the strength properties of rape strawboards also showed a significant improvement allowing for the classification of NC-modified panels as P1 boards, while the non-modified variant did not meet the requirements of the standard. Therefore, it can be concluded that reinforcement of UF adhesive with NC led to the production of strawboards achieving the same strength properties as particleboards bonded with non-modified adhesive. The reason for such an increase in mechanical properties could be the improvement in ductility, fracture energy, and fracture toughness of adhesive bonds [91]. It may be possible due to the large specific surface area of NC, which causes great interfacial interactions between the nanocellulose and the UF resin matrix. The reinforcing effect could be obtained due to the strong intra- and intermolecular hydrogen bonds with the matrix resulting from the high aspect ratio of NC [92]. Moreover, according to Ng et al. [93], the strong bonding ability that can be observed between the nanocellulose and the polymer matrix could allow for the improvement in the transfer of stresses. A similar effect was observed by Dukarska [94] who also noted an improvement in the mechanical properties of rape strawboards allowing for their classification as P1 boards due to the reinforcement of UF adhesive with APTES-modified nano-SiO_2_. The results of thickness swelling were affected only by the material used for the production of boards, not by the application of NC-modified UF adhesive. Rape strawboards were characterized by significantly higher values of thickness swelling compared to particleboards, which could be caused by poorer adhesion resulting in the formation of weaker glue bonds in the manufactured panel [95]. Moreover, no changes in formaldehyde emission were found in the case of reference variants or those containing non-modified NC. The reduction in the HCHO emission was observed only in the case of using APTES-functionalized nanoparticles due to the introduction of amine groups, which, according to Park and Jeong [96], can easily react with both free formaldehyde in the UF adhesive and the hydrolyzed formaldehyde in the obtained panel.

## 4. Conclusions

- The effectiveness of the applied method of nanocellulose silanization is confirmed on the basis of the coarse FTIR spectra indicating the presence of amino groups and increased nitrogen and silicon content.
- The functionalization of nanocellulose with APTES causes a slight decrease in its crystallinity.
- The addition of non-modified nanocellulose does not show the ability to absorb formaldehyde from an aqueous solution. In contrast, in the case of silanized nanocellulose, the formaldehyde-scavenging ability can be observed.
- The presence of APTES-modified nanocellulose in the cured UF adhesive can be observed in the FTIR spectra and increased silicon content.
- The introduction of non-modified NC does not influence the amount of formaldehyde contained in the cured UF adhesive; however, the silanization of nanoparticles prior to homogenization significantly decreased the content of formaldehyde due to the reactions with amino groups contained in the APTES molecule.
- The addition of nanocellulose does not affect the density and thickness swelling of the rape strawboards and particleboards.
- Nanocellulose addition to the UF adhesive causes an improvement in the bending strength, modulus of elasticity, and internal bond of strawboard and particleboard allowing for an extension in the range of their application.
- Functionalization of nanocellulose with APTES leads to a decrease in formaldehyde emission from boards, which is not observed in the case of non-modified nanocellulose.

## Figures and Tables

**Table 1:** Examples of studies concerning UF resin modification with cellulosic particles.

Type of Wood-Based Panel	Cellulose Modification	Effect on Formaldehyde	Additional Effects	References
Plywood	-	No clear effect	Improved bonding quality and mechanical properties Reduced adhesive consumption	[17,18]
Laminated veneer lumber (LVL)	-	No clear effect	Reduced VOC emission Improved bonding quality	[19,20]
Particleboard	-	Decreased emission	Improved mechanical properties Reduced water uptake	[21]
Fiberboard	-	Decreased emission	Improved mechanical properties	[22]
Plywood	Silanization	Decreased emission	Improved bonding quality	[23]
Plywood	Silanization	Decreased emission	Improved bonding quality	[24]
Fiberboard	Silanization	Decreased emission	Improved mechanical properties	[24]
Fiberboard	Silanization	Decreased emission	Improved mechanical properties Reduced water uptake	[25]

**Table 2:** The compositions of adhesive mixtures.

Variant Label	UF Resin (g)	Suspension (g)		Nanocellulose Modification *	H_2_O (g)	Hardener (g)
		H_2_O	Nanocellulose			
REF	100	0	0	-	13.5	2
1%NC-S	100	9	1	S	0	2
2%NC-S	100	18	2	S	0	2
1%NC-N	100	9	1	N	0	2
2%NC-N	100	18	2	N	0	2

* S represents silanized nanocellulose, N represents non-modified nanocellulose.

**Table 3:** Crystallinity of nanocellulose.

Variant	TCI (A1370/A2900)	LOI (A1430/898)
NC	1.13	1.57
NC-S	1.04	1.55

**Table 4:** Elemental composition of nanocellulose.

Variant	Elemental Composition	
	N (%)	Si (mg/kg)
NC	0.00	0.00
NC-S	3.24 ± 0.059	459.13 ± 1.16

**Table 5:** Silicon content in the cured UF adhesive.

Silicon Content (%)				
REF	1%NC-S	2%NC-S	1%NC-N	2%NC-N
0.00 a	0.98 ± 0.02 b	1.99 ± 0.03 c	0.00 a	0.00 a

**Table 6:** Properties of manufactured boards.

Variant of Board	Density (kg/m^3^)	Bending Strength (N/mm^2^)	Modulus of Elasticity (N/mm^2^)	Internal Bond (N/mm^2^)	Thickness Swelling (%)		HCHO Emission (mg/m^2^ h)
					2 h	24 h	
PB-REF	602 ± 12 a	12.4 ± 1.1 b	1582 ± 21 a	0.27 ± 0.03 b	23.1 ± 2.1 a	27.3 ± 2.1 a	4.9 ± 0.2 b
PB-1%NC-S	598 ± 17 a	14.8 ± 1.3 c	1731 ± 12 b	0.38 ± 0.08 c	23.3 ± 2.4 a	28.2 ± 1.8 a	3.3 ± 0.3 a
PB-2%NC-S	601 ± 11 a	14.5 ± 1.7 c	1743 ± 18 b	0.39 ± 0.04 c	23.4 ± 2.1 a	27.8 ± 1.4 a	3.1 ± 0.2 a
PB-1%NC-N	599 ± 19 a	13.9 ± 1.4 c	1738 ± 16 b	0.37 ± 0.05 c	22.7 ± 1.8 a	27.2 ± 2.3 a	4.7 ± 0.3 b
PB-2%NC-N	603 ± 11 a	14.1 ± 0.9 c	1741 ± 12 b	0.39 ± 0.04 c	23.5 ± 1.7 a	26.9 ± 2.1 a	4.8 ± 0.4 b
RB-REF	609 ± 12 a	10.1 ± 1.3 a	1576 ± 22 a	0.17 ± 0.03 a	28.3 ± 2.1 b	31.4 ± 3.1 b	4.8 ± 0.2 b
RB-1%NC-S	596 ± 10 a	12.9 ± 1.4 b	1581 ± 25 a	0.29 ± 0.04 b	27.8 ± 1.6 b	32.1 ± 2.3 b	3.2 ± 0.3 a
RB-2%NC-S	594 ± 11 a	13.1 ± 0.7 b	1579 ± 16 a	0.31 ± 0.03 b	28.3 ± 1.9 b	31.8 ± 1.9 b	3.1 ± 0.2 a
RB-1%NC-N	604 ± 17 a	12.5 ± 0.9 b	1568 ± 18 a	0.29 ± 0.03 b	27.9 ± 2.2 b	32.5 ± 2.9 b	4.6 ± 0.4 b
RB-2%NC-N	601 ± 14 a	12.4 ± 1.3 b	1563 ± 24 a	0.31 ± 0.02 b	28.2 ± 2.3 b	31.5 ± 1.8 b	4.8 ± 0.3 b

PB—particleboard; RB—rape strawboard; letters a, b, and c mark homogeneous groups in HSD Tukey test.

## Data Availability

The data presented in this study are available on request from the corresponding author.
